# Abdominal symmetry index after reconstruction of traumatic lumbar hernia with dual polypropylene mesh: a comparative CT-based study

**DOI:** 10.1007/s10029-026-03759-8

**Published:** 2026-06-30

**Authors:** Jocielle Santos de Miranda, Patrícia Perola Dantas, Marcia Kiyomi Koike, Manoel de Souza Rocha, Mario Paulo Faro, Abel Hiroshi Fernandes Murakami, Allan Antonelli Meira, Edna Frasson de Souza Montero, Claudio Augusto Vianna Birolini, Sergio Henrique Bastos Damous, Edivaldo Massazo Utiyama

**Affiliations:** 1 Division of Surgical Clinic III, Hospital das Clínicas, Faculdade de Medicina da Universidade de São Paulo (HCFMUSP), São Paulo, SP Brazil; 2 Department of Radiology and Oncology, Faculdade de Medicina da Universidade de São Paulo (FMUSP), São Paulo, SP Brazil; 3 Laboratório de Emergências Clínicas (LIM-51), Department of Internal Medicine, Faculdade de Medicina da Universidade de São Paulo (FMUSP), São Paulo, SP Brazil; 4 Department of Surgery, Laboratório de Investigação Médicas, Faculdade de Medicina da Universidade de São Paulo (FMUSP), São Paulo, SP Brazil; 5 Department of Surgery, Faculdade de Medicina da Universidade de São Paulo (FMUSP), São Paulo, SP Brazil

**Keywords:** Traumatic lumbar hernia, Abdominal wall reconstruction, Abdominal symmetry, Computed tomography, Surgical mesh, Lateral abdominal wall

## Abstract

**Purpose:**

Traumatic lumbar hernia (TLH) is a rare and challenging abdominal wall defect. In these patients, treatment success is usually assessed by recurrence, although persistent postoperative bulging or asymmetry may remain clinically relevant even without true failure of repair. This study evaluated the abdominal symmetry index (ASI), defined as a CT-based side-to-side ratio of lateral abdominal wall distance, as an objective morphologic parameter after TLH reconstruction with dual polypropylene mesh.

**Methods:**

A single-center retrospective comparative study was performed at a tertiary academic referral center from 2006 to 2025. The study included 25 consecutive eligible patients with TLH who underwent elective open abdominal wall reconstruction with dual polypropylene mesh and 25 controls without abdominal wall disease. In the TLH group, ASI was assessed on CT before and after surgery; controls underwent a single CT-based assessment. The operative repair consisted of deep preperitoneal/retroperitoneal reinforcement whenever feasible, muscle reapproximation when possible, and preaponeurotic/onlay reinforcement.

**Results:**

Sex distribution did not differ significantly between groups. Mean age was 42 +/- 13 years in the hernia group and 37 +/- 13 years in controls, while mean body mass index was 31 +/- 4 and 28 +/- 5 kg/m2, respectively (*p* = 0.023). Hernia volume was estimated using the ellipsoid formula and had a median value of 443 cm3 (range, 244–1013 cm3). Clinical follow-up was 62 +/- 47 months, and the interval between surgical repair and the latest postoperative CT scan was 687.3 +/- 712.6 days. No recurrence was observed. Before surgery, the measurement on the hernia side was significantly greater than on the contralateral side (185 +/- 24 mm vs. 144 +/- 12 mm; *p* < 0.0001). After reconstruction, this difference was no longer statistically significant (154 +/- 15 mm vs. 149 +/- 13 mm; *p* = 0.1022). Mean ASI was 1.29 +/- 0.18 preoperatively, 1.03 +/- 0.07 postoperatively, and 0.99 +/- 0.03 in controls (overall *p* < 0.0001). Holm-Sidak adjusted pairwise comparisons showed significant differences between preoperative TLH and controls (*p* = 1.14 × 10^-13) and between preoperative and postoperative TLH (*p* = 1.64 × 10^-11), whereas postoperative TLH did not differ significantly from controls (*p* = 0.2142).

**Conclusion:**

ASI was feasible as an exploratory CT-based morphologic index for postoperative evaluation of TLH repair. Reconstruction with dual polypropylene mesh was associated with substantial improvement in abdominal wall symmetry, with postoperative values approaching those of controls. Further validation, including interobserver reproducibility and correlation with patient-reported outcomes, is required before broader adoption.

## Introduction

Traumatic lumbar hernia (TLH) is an uncommon abdominal wall injury usually related to high-energy blunt trauma, particularly motor vehicle collisions, deceleration mechanisms, and direct compression of the abdominal wall [1–6]. Seat-belt-related deceleration injuries are especially relevant because tangential and compressive forces may be transmitted across the lower lateral abdominal wall and iliac crest region. Because of its rarity and variable presentation, diagnosis may be delayed or initially overlooked, despite the well-recognized role of computed tomography in detection and characterization [2, 3, 6].

TLH belongs to the spectrum of abdominal wall disruption after blunt trauma, a condition systematically described and graded by Dennis et al. [4]. This classification helps characterize the severity of abdominal wall injury and provides an anatomical framework for traumatic flank and lumbar defects.

TLH poses a distinct reconstructive challenge. Unlike many midline ventral defects, traumatic lumbar hernias arise in a complex anatomic region characterized by muscular disruption, lateral wall asymmetry, tissue loss, denervation, deficient fascial support, and proximity to rigid osseous landmarks [3–5, 7–10]. The inferior lumbar triangle is a relevant zone of anatomical weakness in this setting and may be more vulnerable than the superior lumbar triangle during high-energy lower lateral abdominal trauma. As a result, successful treatment requires more than simple defect closure.

Traditional outcomes in abdominal wall reconstruction are usually centered on recurrence and perioperative morbidity. However, in lateral abdominal wall defects, particularly traumatic ones, residual bulging or persistent contour deformity may occur even in the absence of true recurrence [11, 12]. This distinction is clinically relevant because a patient may remain dissatisfied or functionally limited despite an anatomically intact repair. In the present study, however, patient satisfaction and hernia-specific function were not primary endpoints, and postoperative quality of life was assessed only with the generic SF-36 questionnaire in a subgroup of patients.

Abdominal wall symmetry is therefore a potentially important but underexplored endpoint in TLH reconstruction. Computed tomography offers an objective platform for this assessment, allowing quantification of abdominal contour and morphologic asymmetry [9, 10]. To our knowledge, no previous report has described ASI for CT-based assessment of symmetry after traumatic lumbar hernia reconstruction. In this manuscript, ASI is presented as an original exploratory morphologic index and not as a previously validated biomarker.

The aim of this study was to assess abdominal wall symmetry after reconstruction of traumatic lumbar hernia using ASI. The study was not designed to demonstrate superiority of this repair over other techniques. We hypothesized that surgical repair would significantly improve symmetry and bring postoperative ASI values closer to those observed in individuals without abdominal wall disease.

## Methods

### Study design and setting

This was a single-center retrospective comparative study conducted at a tertiary academic referral center. Consecutive eligible patients were included from 2006 to 2025.

### Study population

The study included 50 individuals allocated into two groups: a TLH group (*n* = 25) and a control group (*n* = 25). The TLH group comprised all consecutive eligible adult patients with traumatic lumbar hernia who underwent reconstruction using a standardized open dual-mesh technique and had available preoperative and postoperative CT scans. The control group included patients without abdominal wall abnormalities who had abdominal CT scans suitable for morphologic analysis.

Controls were adults who underwent abdominal CT at the same institution for conditions or suspected conditions not involving the abdominal wall or abdominal volume, such as suspected nephrolithiasis. The control group was used as an anatomical reference sample rather than as a perfectly matched comparator.

### Eligibility criteria

Patients were eligible for the TLH group if they had traumatic lumbar hernia and available CT imaging suitable for preoperative and postoperative evaluation. Controls were eligible if they had no abdominal wall disease and adequate CT imaging for analysis.

Control patients were excluded if they had previous abdominal wall surgery, abdominal scars, tissue loss, hernias, tumors, hematomas, fluid collections, inflammatory changes, or any other condition that could alter abdominal wall contour. Additional exclusion criteria for controls included severe neoplastic disease, carcinomatosis, sarcoma, ascites, severe liver disease, immunodeficiency, collagen disease, and inadequate imaging. Patients with inadequate imaging for analysis were also excluded. Cases with inadequate imaging for symmetry assessment were excluded from analysis.

### Ethical aspects

The study was approved by the local institutional research ethics committee (approval number 5.153.664). The study was also registered at the Brazilian Registry of Clinical Trials (ReBEC) under Universal Trial Number U1111-1307-5212 and on Plataforma Brasil under number 53700321.0.0000.0068.

### CT-based assessment of abdominal symmetry

Abdominal wall symmetry was assessed using axial CT images. CT scans were obtained in the supine position during maximal inspiration, with 1.25-mm slices, and measurements were performed using OsiriX Lite version 14.0.1. A vertical line corresponding to the sagittal midline was used to divide the abdomen into right and left hemifields. The sagittal midline was defined using the vertebral body, spinal canal, and spinous process axis. The distance from the midline to the lateral abdominal wall contour was measured bilaterally. In the TLH group, the affected side and the contralateral normal side were assessed before and after surgery. In the control group, bilateral CT-based measurements were used to calculate a reference symmetry index.

The axial CT slice selected for ASI measurement corresponded to the level of maximal deformity. The same anatomical level was used for the preoperative and postoperative analyses in each patient to ensure comparability.

**Fig. 1:**
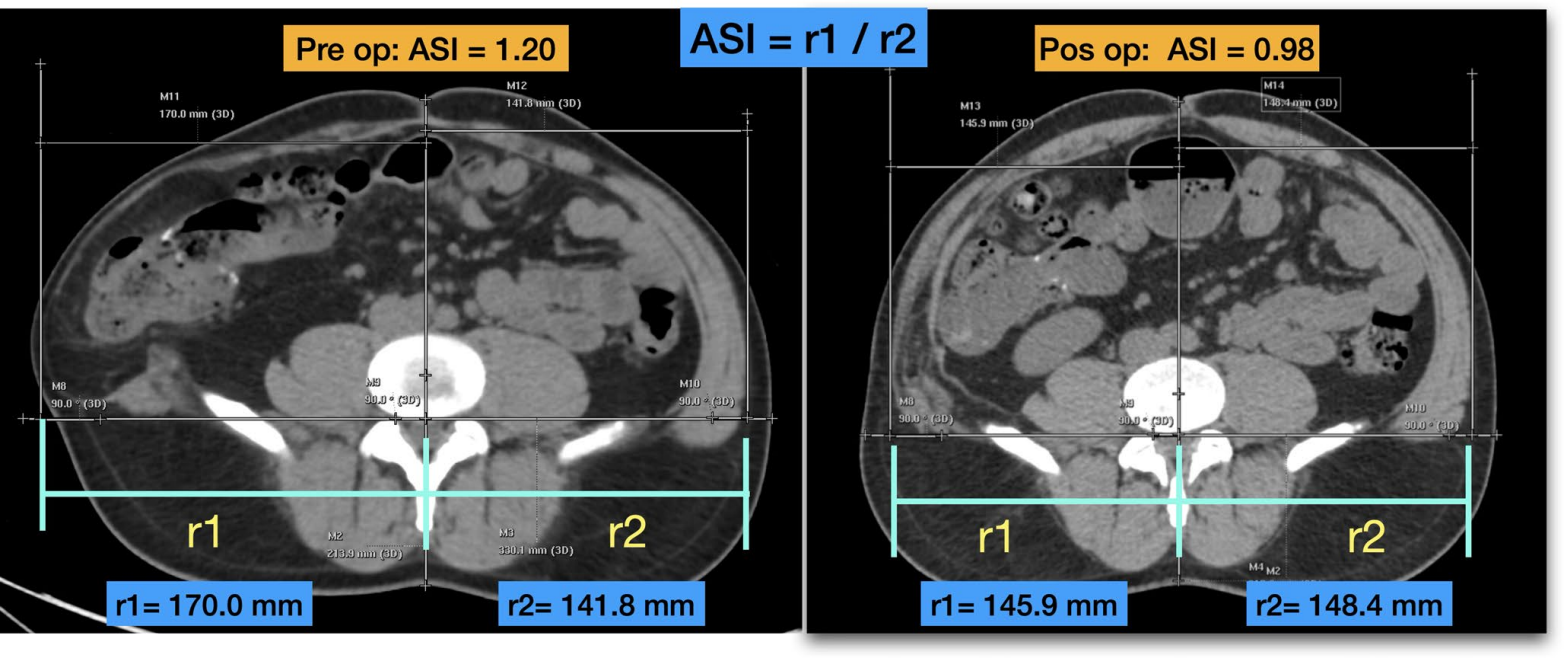
Method for abdominal symmetry index (ASI) calculation on axial computed tomography. The distance from the sagittal midline to the lateral abdominal wall contour was measured bilaterally (r1 and r2), and ASI was calculated as r1/r2. In the TLH group, ASI was calculated as the hernia-side distance divided by the contralateral normal-side distance. In the control group, ASI was calculated as the right-side distance divided by the left-side distance. The preoperative image illustrates abdominal asymmetry, whereas the postoperative image shows restoration toward symmetry

The abdominal symmetry index (ASI) was defined as the ratio between the distance measured on the hernia side (numerator) and the corresponding distance measured on the contralateral normal side (denominator). In the control group, ASI was calculated as the right-side distance divided by the left-side distance (r1/r2), rather than as the larger side divided by the smaller side. Measurements were obtained on axial CT using the sagittal midline as the central reference. Values closer to 1 indicate greater symmetry. In the TLH group, ASI was measured preoperatively and postoperatively; in controls, it was measured once for comparison. The method used for ASI calculation is illustrated in Fig. 1.

The CT measurements were performed by one radiologist. Interobserver reliability was not assessed in the present study.

### Additional assessment

The study also included postoperative quality-of-life assessment using the SF-36 questionnaire in a subgroup of patients [13]. Because the primary aim of this manuscript was morphologic assessment of repair quality, SF-36 findings were treated as supportive secondary data. SF-36 was not available preoperatively and is not a hernia-specific or body-image-specific instrument.

An exploratory correlation analysis was performed between percentage change in ASI from the preoperative to postoperative period and postoperative SF-36 domains in patients with available SF-36 data. Percentage change in ASI was calculated as: (postoperative ASI - preoperative ASI) / preoperative ASI x 100.

### Surgical technique

All patients in the TLH group underwent elective open lateral abdominal wall reconstruction with dual polypropylene mesh. Surgery was performed with the patient in contralateral lateral decubitus. The defect was exposed through a lumbar incision, followed by wide dissection of the planes, development of the preperitoneal or retroperitoneal space when necessary, reduction of hernia contents, and treatment of fibrosis or foreign material when present. The peritoneum was plicated with absorbable suture when required.

A heavyweight macroporous polypropylene mesh was placed deeply in the preperitoneal/retroperitoneal plane whenever feasible and fixed with six trans-fascial U stitches using absorbable polyglactin 910. Non-absorbable sutures and bone anchors were not used for prosthetic fixation. No mesh was used as a true inlay sutured only to the edges of the defect.

In some patients, broad dissection with the usual overlap was limited by tissue loss, dense fibrosis, or complex pelvic fracture with bony callus involving the iliac crest. In these cases, reinforcement was still performed with mesh overlap; the smallest overlap was 6 cm in two cases. In all other cases, the standard overlap was at least 12 cm. This was followed by muscular reconstruction whenever reconstructible tissue was present. A second polypropylene mesh was then positioned in the preaponeurotic plane as an onlay reinforcement with drainage.

### Hernia characterization

Hernia and abdominal wall injury characteristics were described according to the grading system proposed by Dennis et al. [4]. The affected abdominal wall muscles were also recorded.

Hernia volume was not obtained by direct CT/DICOM volumetry. It was estimated using the ellipsoid formula based on CT-derived measurements of defect width, height, and length: volume = width x height x length x pi/6.

In response to anatomical review of the cohort, lumbar triangle involvement was also assessed on CT and classified as inferior lumbar triangle involvement, superior lumbar triangle involvement, or combined involvement.

### Variables collected

Baseline demographic variables included age, sex, and body mass index. Available comorbidities and smoking history were also collected. Hernia-related variables included laterality, defect characteristics, mechanism of trauma, and hernia volume. Operative and postoperative variables included drain use, hospitalization, complications, recurrence, and follow-up duration. Imaging outcomes included absolute wall measurements and ASI. Postoperative SF-36 scores were collected as supportive secondary data in a subgroup of patients.

### Outcomes

The primary outcome was change in ASI after TLH reconstruction.

Secondary outcomes included postoperative complications, surgical site occurrence, recurrence, length of hospital stay, follow-up duration, and postoperative SF-36 results. Postoperative bulging was also assessed clinically during follow-up.

### Statistical analysis

Data were analyzed according to variable type and distribution. Categorical variables were expressed as absolute and relative frequencies and compared using the chi-square test. Continuous variables were reported as mean +/- standard deviation or median and range, as appropriate. Normality was assessed before inferential testing. Comparisons between independent groups were performed using Student’s t test for normally distributed variables and the Mann-Whitney test for non-normally distributed variables. Paired comparisons between preoperative and postoperative measurements were performed using the paired Student’s t test. The comparison of ASI among controls, preoperative TLH, and postoperative TLH was performed using one-way analysis of variance followed by the Holm-Sidak test for multiple comparisons. A two-sided p value < 0.05 was considered statistically significant.

**Fig. 2:**
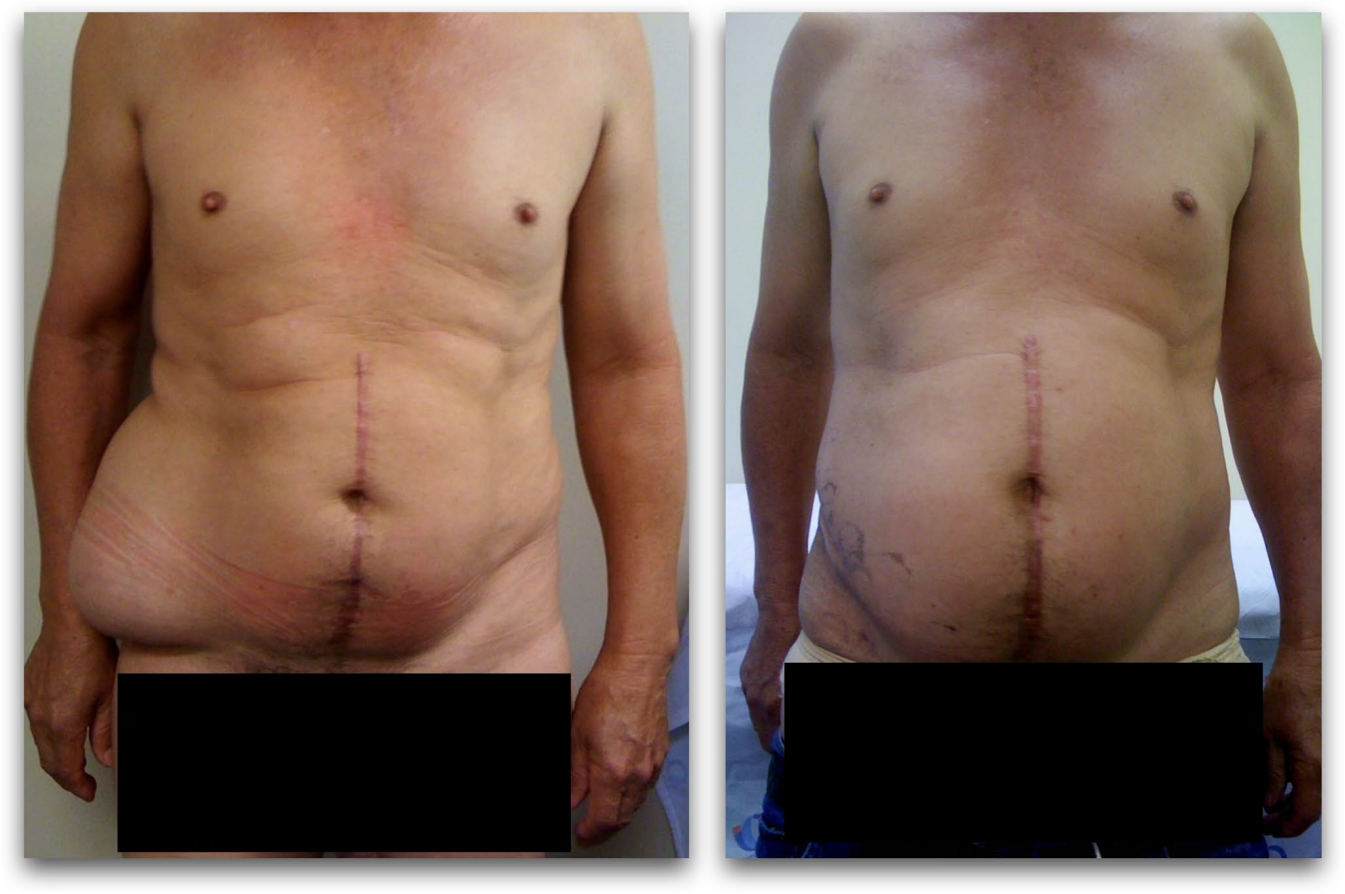
Representative preoperative and postoperative clinical photographs demonstrating improvement in abdominal wall contour and symmetry after reconstruction of traumatic lumbar hernia with dual polypropylene mesh

Because BMI differed significantly between groups at baseline, an exploratory adjusted analysis was conducted using BMI as a covariate. This analysis was performed to evaluate whether the association between group status and ASI remained consistent after accounting for BMI imbalance. The control group was interpreted as an anatomical reference group rather than as a fully matched comparator. The paired preoperative-to-postoperative analysis within the TLH group was considered the principal comparison for treatment-related change (Fig. 2).

## Results

### Baseline characteristics

**Fig. 3:**
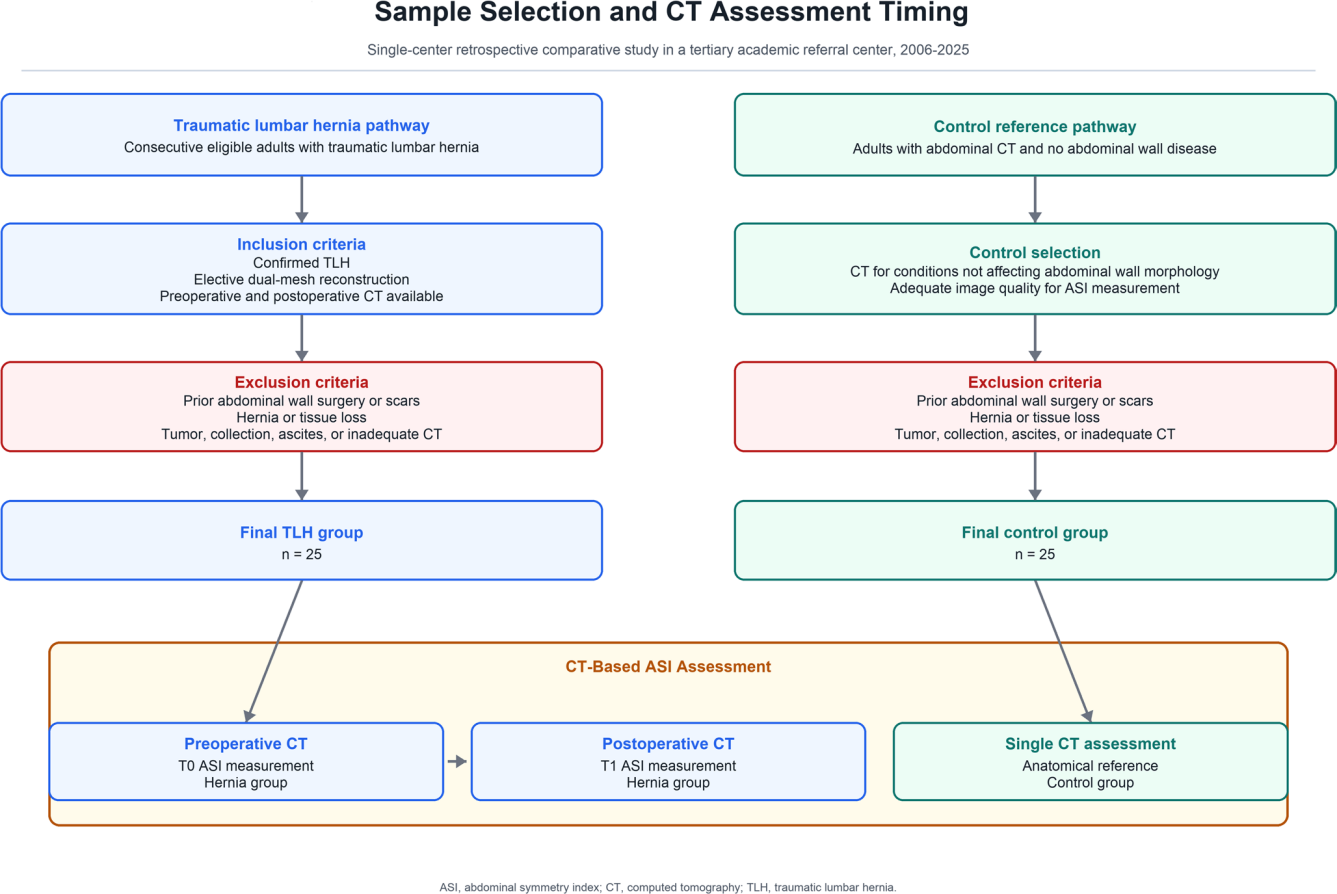
Revised study flow diagram showing the source population, eligibility criteria, exclusions, final TLH group, final control group, and timing of CT assessments. In the TLH group, abdominal symmetry was evaluated preoperatively and postoperatively. In the control group, a single assessment was performed as the anatomical reference

A total of 50 individuals were included, 25 in the TLH group and 25 in the control group. The final analytical sample comprised 25 patients in the TLH group and 25 individuals in the control group, as shown in Fig. 3. Sex distribution did not differ significantly between groups (*p* = 0.39). In the control group, 12 patients (48%) were male and 13 (52%) were female, whereas in the TLH group, 15 patients (60%) were male and 10 (40%) were female. Mean age was 42 +/- 13 years in the hernia group and 37 +/- 13 years in controls (*p* = 0.1730). Body weight tended to be higher in the hernia group than in controls (87 +/- 18 vs. 78 +/- 14 kg; *p* = 0.054). BMI was significantly higher in the hernia group compared with controls (31 +/- 4 vs. 28 +/- 5 kg/m2; *p* = 0.023).

Available comorbidities and smoking history in the TLH group are summarized in Table 1. Systemic arterial hypertension was present in 7 patients (28%), diabetes mellitus in 2 (8%), hypothyroidism in 1 (4%), asthma in 1 (4%), and bipolar disorder with schizoaffective disorder in 1 (4%); 13 patients (52%) had no specified comorbidity. Eleven patients (44%) had never smoked, 5 (20%) had smoked for up to 5 years, 6 (24%) for 5–10 years, and 3 (12%) for 10–20 years.

**Table 1:** Baseline characteristics of the study population

Variable	Control group (*n* = 25)	TLH group (*n* = 25)	*p* value
Male sex, n (%)	12 (48%)	15 (60%)	0.39
Female sex, n (%)	13 (52%)	10 (40%)	
Age, years	37 +/- 13	42 +/- 13	0.1730
Weight, kg	78 +/- 14	87 +/- 18	0.054
BMI, kg/m2	28 +/- 5	31 +/- 4	0.023
Systemic arterial hypertension, n (%)	Not assessed	7 (28%)	
Diabetes mellitus, n (%)	Not assessed	2 (8%)	
Hypothyroidism, n (%)	Not assessed	1 (4%)	
Asthma, n (%)	Not assessed	1 (4%)	
Bipolar disorder with schizoaffective disorder, n (%)	Not assessed	1 (4%)	
No specified comorbidity, n (%)	Not assessed	13 (52%)	
Never smoked, n (%)	Not assessed	11 (44%)	
Smoking history up to 5 years, n (%)	Not assessed	5 (20%)	
Smoking history 5–10 years, n (%)	Not assessed	6 (24%)	
Smoking history 10–20 years, n (%)	Not assessed	3 (12%)	

Data are presented as mean +/- standard deviation or n (%). Comorbidity and smoking history were available for the TLH group and were not assessed in the control group. BMI, body mass index; TLH, traumatic lumbar hernia

### Hernia characteristics

The median hernia volume was 443 cm3 (range, 244–1013 cm3), reflecting the complexity of the defects analyzed. Additional defect characteristics, including laterality, classification, muscle involvement, timing of diagnosis, trauma mechanism, and lumbar triangle involvement, are summarized in Table 2. Group allocation and timing of assessments are shown in Fig. 3.

**Table 2:** Hernia characteristics in the traumatic lumbar hernia group

Variable	TLH group (*n* = 25)
Right side, n (%)	16 (64%)
Left side, n (%)	9 (36%)
Dennis grade III, n (%)	2 (8%)
Dennis grade IV, n (%)	3 (12%)
Dennis grade V, n (%)	20 (80%)
Width, cm	7 +/- 3
Height, cm	10 (7–13)
Length, cm	8 (7–12)
Volume, cm3	443 (244–1013)
External oblique involvement, n (%)	19 (76%)
Internal oblique involvement, n (%)	19 (76%)
Transversus abdominis involvement, n (%)	20 (80%)
Rectus abdominis involvement, n (%)	2 (8%)
Quadratus lumborum involvement, n (%)	1 (4%)
Inferior lumbar triangle involvement, n (%)	25 (100%)
Superior lumbar triangle involvement in continuity with inferior triangle injury, n (%)	2 (8%)
Diagnosis during hospitalization, n (%)	3 (12%)
Diagnosis during initial trauma assessment, n (%)	9 (36%)
Diagnosis during outpatient follow-up, n (%)	13 (52%)
Motor vehicle collision, n (%)	13 (52%)
Motorcycle accident, n (%)	7 (28%)
Fall from height, n (%)	3 (12%)
Pedestrian struck, n (%)	2 (8%)
Seat belt mechanism, n (%)	12 (48%)
Direct compression, n (%)	8 (32%)
Ejection, n (%)	3 (12%)
Crushing, n (%)	2 (8%)

Continuous variables are presented as mean +/- standard deviation or median (range), according to distribution. Hernia and abdominal wall injury characteristics were described according to the grading system proposed by Dennis et al. [4]. Hernia volume was estimated using the ellipsoid formula: width x height x length x pi/6

**Table 3:** Operative and postoperative outcomes

Variable	TLH group (*n* = 25)
Elective repair, n (%)	25 (100%)
Time from diagnosis to repair, days	21 (9–62)
Postoperative time to discharge, days	4 (3–5)
Hospital stay, days	6 (5–8)
Drain use duration, days	8 (5–14)
Operative duration, min	223 (200–254)
Anesthesia duration, min	290 (250–364)
Clinical follow-up, months	62 +/- 47
Interval from repair to latest postoperative CT, days	687.3 +/- 712.6; median 351 (5-2122)
Recurrence, n (%)	0 (0%)
Postoperative bulging, n (%)	0 (0%)
No complications, n (%)	17 (68%)
Seroma treated by puncture, n (%)	3 (12%)
Surgical site infection requiring drainage, n (%)	1 (4%)
Intermuscular hematoma associated with seroma, n (%)	1 (4%)
Wound opening treated with negative-pressure therapy, n (%)	1 (4%)
Extensive flank ecchymosis, n (%)	1 (4%)
Wound infection with fistula requiring mesh exchange/explantation, n (%)	1 (4%)
Clavien-Dindo I, n (%)	1 (4%)
Clavien-Dindo IIIa, n (%)	5 (20%)
Clavien-Dindo IIIb, n (%)	2 (8%)
Clavien-Dindo IV-V, n (%)	0 (0%)
Chronic mesh infection requiring reoperation, n (%)	1 (4%)

Complications were grouped according to the Clavien-Dindo classification, using the highest grade per patient for grouped severity. Continuous variables are presented as mean +/- standard deviation or median (range), according to distribution

All 25 patients had involvement of the inferior lumbar triangle, and 2 patients had associated superior lumbar triangle involvement in continuity with the inferior lumbar triangle injury.

### Operative and postoperative outcomes

All repairs were performed electively. Median hospital stay was 6 days (range, 5–8 days). Mean postoperative clinical follow-up was 62 +/- 47 months. The interval between surgical repair and the latest available postoperative CT scan was 687.3 +/- 712.6 days, with a median of 351 days and a range from 5 to 2122 days. No recurrence was observed during follow-up. No postoperative bulging was reported in the operated patients during follow-up.

Postoperative complications are detailed in Table 3. Seventeen patients (68%) had no postoperative complications. The most frequent complication was seroma treated by puncture in 3 patients (12%). According to the highest Clavien-Dindo grade per patient, 1 patient (4%) had grade I complications, 5 (20%) had grade IIIa complications, and 2 (8%) had grade IIIb complications. No grade IV or V complications were observed.

One patient (4%) developed chronic mesh infection requiring reoperation. In this patient, only the deep preperitoneal mesh was removed. The onlay mesh over the muscle was preserved because it was well incorporated. The patient underwent re-reconstruction in the same preperitoneal space with a flat mesh and wide drainage using 19-Fr Blake drains. There were no complications related to the reoperation. The patient was followed until 2022, for 85 months, and an abdominal CT scan performed in 2022 showed no signs of recurrence.

### Primary outcome: abdominal symmetry

Before surgery, the absolute measurement on the hernia side was significantly greater than on the normal contralateral side (185 +/- 24 mm vs. 144 +/- 12 mm; *p* < 0.0001). After reconstruction, this difference was no longer statistically significant (154 +/- 15 mm vs. 149 +/- 13 mm; *p* = 0.1022). In longitudinal analysis, the hernia side showed a significant reduction after surgery (*p* = 0.0065), whereas the contralateral side remained stable (*p* = 0.0576).

Mean ASI was 1.29 +/- 0.18 in the preoperative TLH group, 1.03 +/- 0.07 in the postoperative TLH group, and 0.99 +/- 0.03 in the control group. The overall comparison among groups was significant (*p* < 0.0001). Holm-Sidak adjusted pairwise comparisons showed that ASI was significantly higher in the preoperative TLH group than in controls (*p* = 1.14 × 10^-13) and significantly decreased after repair (preoperative TLH vs. postoperative TLH, *p* = 1.64 × 10^-11). Postoperative TLH ASI did not differ significantly from the control group (*p* = 0.2142).

After adjustment for BMI, the main ASI findings remained consistent with the unadjusted analysis, suggesting that the observed group difference was not fully explained by baseline BMI imbalance.

In the control group, bilateral measurements were reviewed across three CT levels. When the mean of the three levels was calculated for each patient, right-side and left-side distances were 127.8 +/- 10.8 mm and 128.8 +/- 10.0 mm, respectively (paired *p* = 0.2111), with a mean right/left ASI of 0.992 +/- 0.031.

**Fig. 4:**
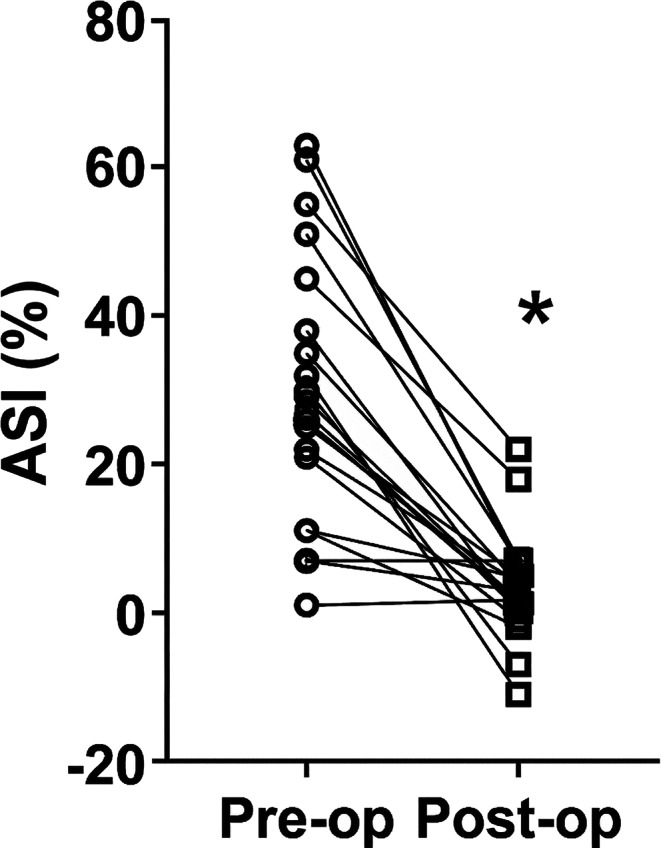
Preoperative and postoperative abdominal symmetry index (ASI) values in patients with traumatic lumbar hernia. Individual paired ASI values are shown for the preoperative and postoperative periods. The previous panel 4B was removed. Statistical comparison between preoperative and postoperative measurements was performed using the paired Student’s t test. Postoperative ASI values were significantly lower than preoperative values (Holm-Sidak adjusted *p* = 1.64 × 10^-11 for preoperative vs. postoperative TLH in the three-group ASI comparison)

Representative CT-based ASI measurement and clinical contour improvement are shown in Figs. 1 and 2. The reduction in ASI from the preoperative to the postoperative period is illustrated in Fig. 4. CT-based symmetry outcomes are summarized in Table 4.

**Table 4:** CT-based abdominal wall symmetry outcomes

Variable	Preoperative TLH	Postoperative TLH	Control group	*p* value
Normal side, mm	144 +/- 12	149 +/- 13	-	0.0576
Hernia side, mm	185 +/- 24	154 +/- 15	-	0.0065
Postoperative normal side vs. hernia side, mm	-	149 +/- 13 vs. 154 +/- 15	-	0.1022
Control right side, mm	-	-	127.8 +/- 10.8	0.2111*
Control left side, mm	-	-	128.8 +/- 10.0	
ASI	1.29 +/- 0.18	1.03 +/- 0.07	0.99 +/- 0.03	< 0.0001
ASI: preoperative TLH vs. control				1.14 × 10^-13
ASI: preoperative TLH vs. postoperative TLH				1.64 × 10^-11
ASI: postoperative TLH vs. control				0.2142

Data are presented as mean +/- standard deviation. ASI, abdominal symmetry index; TLH, traumatic lumbar hernia. ASI comparisons among control, preoperative TLH, and postoperative TLH groups were performed using one-way ANOVA followed by the Holm-Sidak multiple-comparisons test. *Paired comparison between right-side and left-side control measurements, calculated after averaging the three CT levels for each control patient

Patients with minimal ASI change were reviewed. The variables most related to this observation were lower baseline ASI and Dennis grade III injury, particularly when baseline ASI represented less than a 10% side-to-side difference. No statistical correlation was observed with other variables evaluated.

### Quality of life and ASI/SF-36 correlation

Postoperative SF-36 was available for 15 patients. Scores were highest for physical functioning (88.1 +/- 4.61), mental health (84.1 +/- 4.10), and social functioning (78.1 +/- 4.00). Lower scores were observed for vitality (54.7 +/- 3.81) and role physical (58.6 +/- 4.19). All SF-36 domain scores are shown in Table 5. These findings suggest preserved global functional and psychosocial recovery despite some residual limitations in physical recovery domains.

**Table 5:** Postoperative SF-36 results in the TLH group

SF-36 domain	Mean +/- SD
Physical functioning	88.1 +/- 4.61
Bodily pain	74.0 +/- 3.82
General health	63.7 +/- 3.96
Vitality	54.7 +/- 3.81
Social functioning	78.1 +/- 4.00
Role emotional	69.1 +/- 4.22
Role physical	58.6 +/- 4.19
Mental health	84.1 +/- 4.10

SF-36 was assessed postoperatively in 15 patients. SF-36, Medical Outcomes Study 36-Item Short-Form Health Survey; TLH, traumatic lumbar hernia

The percentage change in ASI from the preoperative to the postoperative period was not significantly correlated with the mean SF-36 score or with any individual SF-36 domain (Table 6). Pearson’s correlation between ASI percentage change and the mean SF-36 score was weak and not statistically significant (*r* = 0.247; *p* = 0.395). Similarly, Spearman’s rank correlation showed no significant monotonic association (rho = 0.152; *p* = 0.604).

**Table 6:** Correlation between percentage change in ASI and SF-36 domains

SF-36 domain	Pearson *r*	*p* value	Spearman rho	*p* value
Physical functioning	-0.092	0.755	-0.032	0.913
Role limitations due to physical health	0.221	0.449	0.031	0.916
Bodily pain	0.276	0.340	0.337	0.238
General health	0.062	0.833	0.011	0.970
Vitality	0.299	0.300	0.323	0.260
Social functioning	0.258	0.374	0.265	0.361
Role limitations due to emotional problems	0.256	0.377	0.294	0.307
Mental health	0.295	0.306	0.221	0.447
Mean SF-36 score	0.247	0.395	0.152	0.604

Percentage change in ASI was calculated as: (postoperative ASI - preoperative ASI) / preoperative ASI x 100. Negative values indicate reduction of asymmetry, whereas positive values indicate increase in postoperative asymmetry. Correlations were calculated using Pearson’s correlation coefficient and Spearman’s rank correlation coefficient. No statistically significant correlation was observed between ASI percentage change and SF-36 domains. ASI, abdominal symmetry index; SF-36, Short Form-36 Health Survey

## Discussion

The main finding of this study is that reconstruction of traumatic lumbar hernia with dual polypropylene mesh produced a marked objective improvement in abdominal wall symmetry, as demonstrated by CT-based ASI assessment. Postoperative ASI values approached those observed in controls, while the side-to-side difference present before surgery was no longer significant after repair. These findings support the concept that, in TLH, successful reconstruction should not be evaluated by recurrence alone.

The present study does not establish ASI as a validated biomarker. Rather, it proposes ASI as an original exploratory CT-based morphologic index for this specific reconstructive scenario. To our knowledge, no previous report has described this index for symmetry assessment after traumatic lumbar hernia reconstruction. Formal validation, including interobserver reproducibility, external cohorts, and correlation with patient-perceived bulging, body image, hernia-specific quality of life, and functional outcomes, remains necessary. TLH is a rare lesion with unique reconstructive implications [1–8]. Previous studies have emphasized its association with high-energy trauma, delayed or challenging diagnosis, and frequent coexistence with associated abdominal injuries [2–4, 6]. Contemporary reviews and systematic analyses have also highlighted the heterogeneity of presentation and management strategies, as well as the limited quality of available evidence [5, 8]. Within this context, the present study contributes a different perspective by focusing on postoperative morphologic restoration rather than solely on repair durability.

All patients in this series had inferior lumbar triangle involvement, while only 2 patients had associated superior lumbar triangle involvement. This predominance is anatomically plausible because the inferior lumbar triangle is a region of relative weakness and is more exposed to tangential and compressive forces transmitted across the lower lateral abdominal wall and iliac crest region during high-energy deceleration and seat-belt-related trauma.

This distinction is important because complex lateral abdominal wall defects can remain clinically problematic even in the absence of true recurrence. Mesh bulging and persistent contour deformity have already been recognized as relevant negative outcomes in abdominal wall reconstruction [11, 12]. In TLH, where muscle avulsion, asymmetry, and loss of lateral wall integrity are common, this issue may be even more pronounced. A binary definition of success based only on recurrence may therefore fail to capture the actual quality of repair. In the present cohort, no postoperative bulging was reported.

The rationale for ASI is grounded in the concept that the abdominal wall is a dynamic functional unit rather than a passive barrier [9, 10]. Imaging-based approaches have increasingly demonstrated that abdominal wall morphology and function can be objectively assessed using CT [9, 10]. In this study, ASI proved feasible and sensitive to change, showing a clear postoperative shift toward normal values. This suggests that the metric may help quantify morphologic restoration after reconstruction.

The clinical implications are relevant. In practice, postoperative flank fullness or asymmetry may be difficult to interpret, particularly when the distinction between residual bulging and true recurrence is uncertain. An objective index such as ASI may help standardize this assessment, facilitate follow-up, and provide an outcome measure that is more closely aligned with patient-perceived contour restoration. However, in this study ASI was not significantly correlated with SF-36 domains. This absence of correlation should be interpreted cautiously because SF-36 was available only postoperatively, included only 15 patients, and is a generic quality-of-life instrument rather than a hernia-specific or body-image-specific measure.

Although the present manuscript is not intended as a technical paper, the favorable symmetry results likely reflect the reconstructive strategy used. Broad overlap, deep fixation, muscle reapproximation, and superficial reinforcement may all contribute to restoring lateral wall support and contour. This approach is consistent with the broader abdominal wall reconstruction principle that restoration of force transmission and wall geometry is as important as simple defect closure [9, 14, 15]. The absence of postoperative bulging in this series may be related to muscle reinsertion whenever feasible and the association of extensive preperitoneal/retroperitoneal and preaponeurotic prosthetic reinforcement.

The study also demonstrated durable repair, with no recurrence during 62 +/- 47 months of clinical follow-up, and acceptable morbidity considering the complexity of the defects treated. The complication profile was dominated by manageable events such as seroma, although one patient required reoperation because of chronic mesh infection. In that case, only the preperitoneal mesh was removed; the incorporated onlay mesh was preserved. Follow-up for that patient reached 85 months, with abdominal CT in 2022 showing no recurrence. These clinical results support the mechanical effectiveness of reconstruction while reinforcing that the central contribution of this study lies in postoperative morphologic assessment.

The SF-36 findings should be interpreted cautiously, as they were available only postoperatively and in a subset of patients. Nonetheless, the pattern observed was compatible with good overall recovery, particularly in physical functioning, mental health, and social functioning. This supports the concept that repair outcomes are multidimensional and include both anatomical and patient-centered domains [13]. The lack of significant association between ASI percentage change and SF-36 domains does not exclude clinical relevance of symmetry restoration, but indicates that future studies should use hernia-specific quality-of-life and body-image instruments.

Given that BMI was significantly higher in the hernia group, we performed an exploratory BMI-adjusted analysis. The persistence of the main ASI findings after adjustment supports the robustness of the association, although residual confounding cannot be excluded due to the observational design and sample size.

This study has several strengths. It addresses a rare condition in a relatively robust single-center series, includes a control group, uses long-term follow-up, and proposes an objective imaging endpoint directly linked to a relevant clinical problem. At the same time, the study has limitations. Its retrospective design introduces the possibility of selection bias, the sample size is limited by the rarity of the disease, and ASI has not yet undergone external validation or formal interobserver reproducibility testing. The CT measurements were performed by one radiologist, and interobserver agreement was not assessed. The control group was not fully matched for BMI and should be interpreted as an anatomical reference rather than as a perfectly matched comparator. Comorbidity capture was limited by the retrospective design, and postoperative SF-36 data were available only in a subgroup. Future studies should assess the reproducibility of ASI, its correlation with patient-reported body image and hernia-specific outcomes, and its applicability to other lateral abdominal wall defects.

In summary, this study supports a broader concept of success in TLH repair. In these patients, restoration of abdominal wall symmetry is not merely a cosmetic issue, but a relevant morphologic expression of effective reconstruction. ASI appears to be a practical exploratory CT-based parameter that may complement recurrence and complication rates in future studies of complex lateral abdominal wall repair, pending formal validation.

## Conclusion

In traumatic lumbar hernia, successful reconstruction should not be defined by recurrence alone. In this study, dual-mesh abdominal wall reconstruction produced durable repair and substantial correction of abdominal asymmetry, with postoperative abdominal symmetry index values approaching those of controls. ASI is a feasible exploratory CT-based metric that may broaden outcome assessment in complex lateral abdominal wall reconstruction by incorporating an objective measure of morphologic restoration. Further validation is required before routine clinical adoption.

## Data Availability

The datasets generated and/or analyzed during the current study are available from the corresponding author on reasonable request.
